# Supplementary material for: Post-myocardial infarction heart failure and long-term high-fat diet: Cardiac endoplasmic reticulum stress and unfolded protein response in Sprague Dawley rat model
Source: PLoS One. 2024 Sep 18;19(9):e0308833. doi: 10.1371/journal.pone.0308833 (PMC11410228; doi:10.1371/journal.pone.0308833)
Supplement: S1 Raw images — (PDF) [file pone.0308833.s001.pdf]

3-nitrotysine

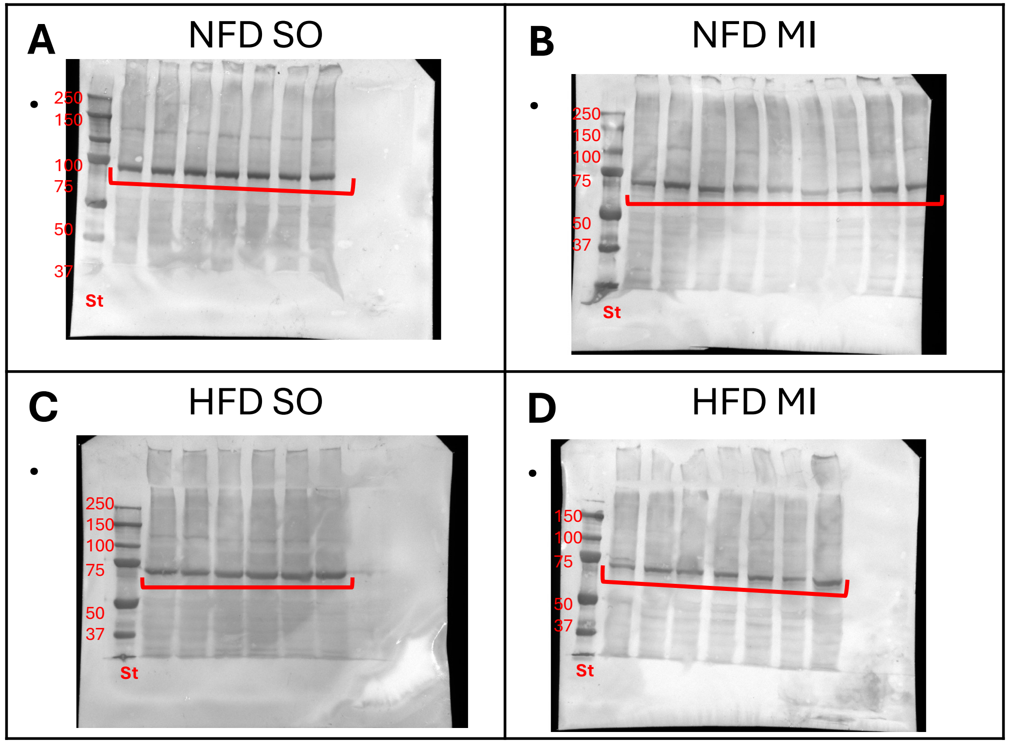

Figure 1. Western Blot results for 3-nitrosine. A. PVDF membrane with left ventricular samples from sham operated rats fed with the normal fat diet (NFD SO). B. PVDF membrane with left ventricular samples from rats with ligation of the coronary artery fed with the normal fat diet (NFD MI). C. PVDF membrane with left ventricular samples from sham operated rats fed with the normal fat diet (HFD SO). D. PVDF membranę with left ventricular samples from rats with ligation of the coronary artery fed with the normal fat diet. St – protein standard.

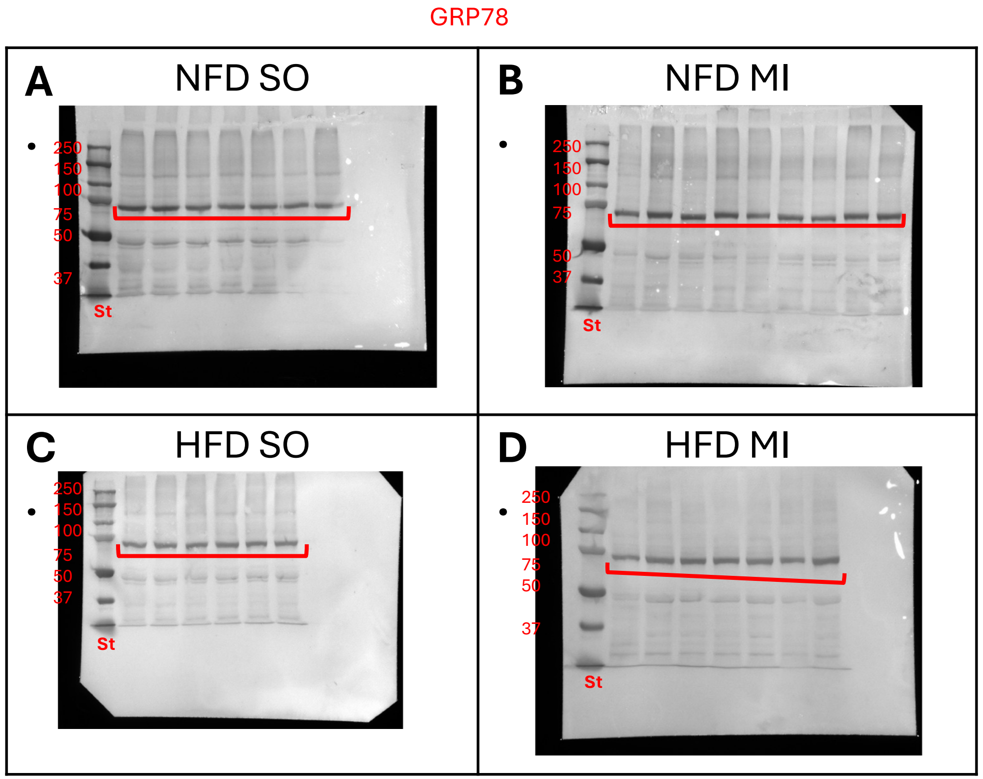

Figure 2. Western Blot results for 78-kDa glucose-regulated protein (GRP78). A. PVDF membrane with left ventricular samples from sham operated rats fed with the normal fat diet (NFD SO). B. PVDF membrane with left ventricular samples from rats with ligation of the coronary artery fed with the normal fat diet (NFD MI). C. PVDF membrane with left ventricular samples from sham operated rats fed with the normal fat diet (HFD SO). D. PVDF membranę with left ventricular samples from rats with ligation of the coronary artery fed with the normal fat diet. St – protein standard.

MPO

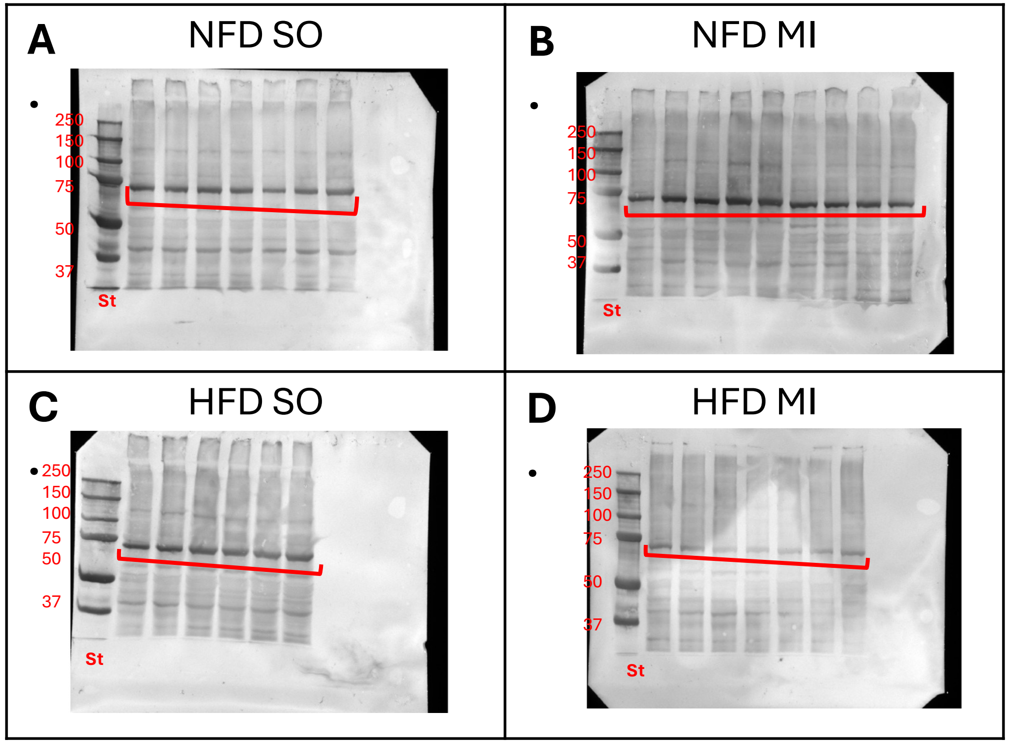

Figure 3. Western Blot results for myeloperoxidase (MPO). A. PVDF membrane with left ventricular samples from sham operated rats fed with the normal fat diet (NFD SO). B. PVDF membrane with left ventricular samples from rats with ligation of the coronary artery fed with the normal fat diet (NFD MI). C. PVDF membrane with left ventricular samples from sham operated rats fed with the normal fat diet (HFD SO). D. PVDF membranę with left ventricular samples from rats with ligation of the coronary artery fed with the normal fat diet. St – protein standard.

IRE1α

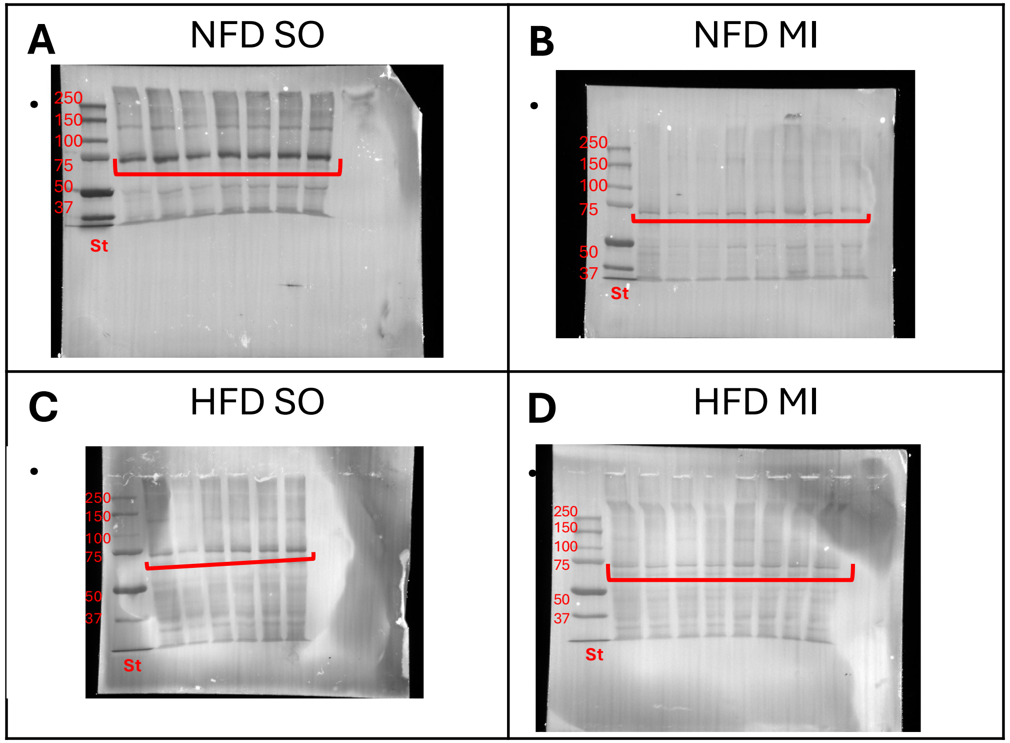

Figure 4. Western Blot results for inositol-requiring enzyme type 1 α (IRE1α). A. PVDF membrane with left ventricular samples from sham operated rats fed with the normal fat diet (NFD SO). B. PVDF membrane with left ventricular samples from rats with ligation of the coronary artery fed with the normal fat diet (NFD MI). C. PVDF membrane with left ventricular samples from sham operated rats fed with the normal fat diet (HFD SO). D. PVDF membranę with left ventricular samples from rats with ligation of the coronary artery fed with the normal fat diet. St – protein standard.

The expected size of the detected IRE1α protein was around 110 kDa. However, in our results, a band was observed at approximately 70 kDa. This discrepancy might be due to differences in protein migration during electrophoresis, post-translational modifications, or protein degradation during sample preparation. We also observed additional bands at sizes other than 70 kDa, which could represent degradation products of IRE1α or non-specific antibody binding. Each sample was normalized to β-actin to minimize these differences, allowing for relative protein level comparisons between samples.

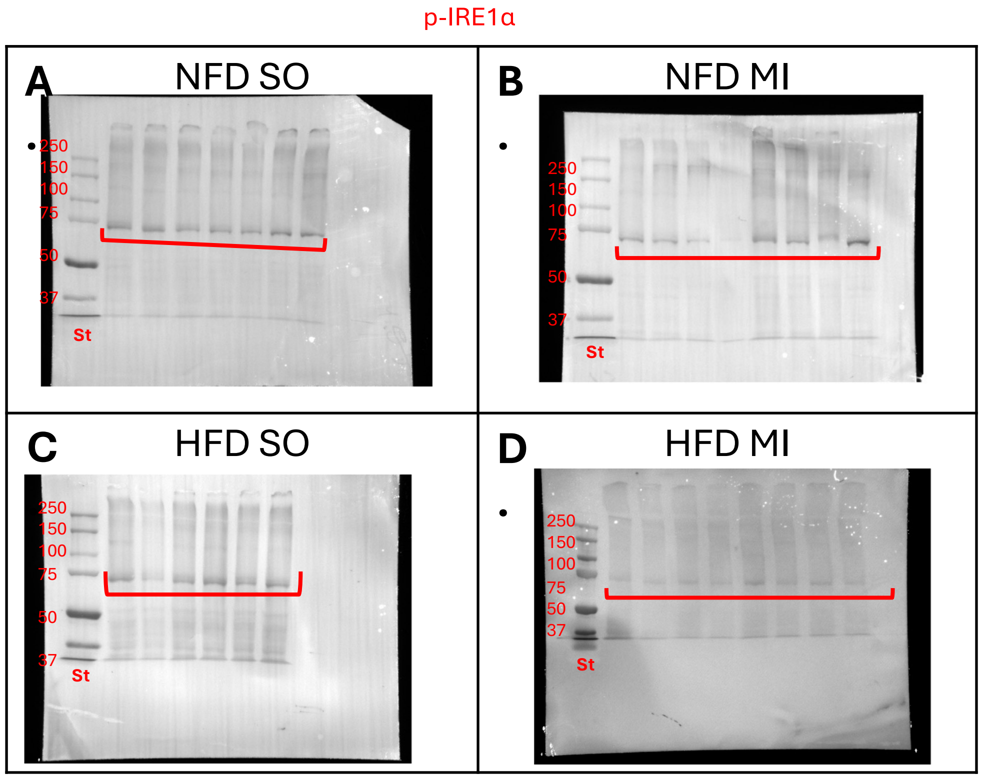

Figure 5. Western Blot results for phospho-IRE1α. A. PVDF membrane with left ventricular samples from sham operated rats fed with the normal fat diet (NFD SO). B. PVDF membrane with left ventricular samples from rats with ligation of the coronary artery fed with the normal fat diet (NFD MI). C. PVDF membrane with left ventricular samples from sham operated rats fed with the normal fat diet (HFD SO). D. PVDF membranę with left ventricular samples from rats with ligation of the coronary artery fed with the normal fat diet. St – protein standard.

The expected size of the detected phospho-IRE1α protein was around 110 kDa. However, in our results, a band was observed at approximately 75 kDa. This discrepancy might be due to differences in protein migration during electrophoresis, post-translational modifications, or protein degradation during sample preparation. We also observed additional bands at sizes other than 75 kDa, which could represent degradation products of phospho-IRE1α or non-specific antibody binding. Each sample was normalized to β-actin to minimize these differences, allowing for relative protein level comparisons between samples.

eNOS

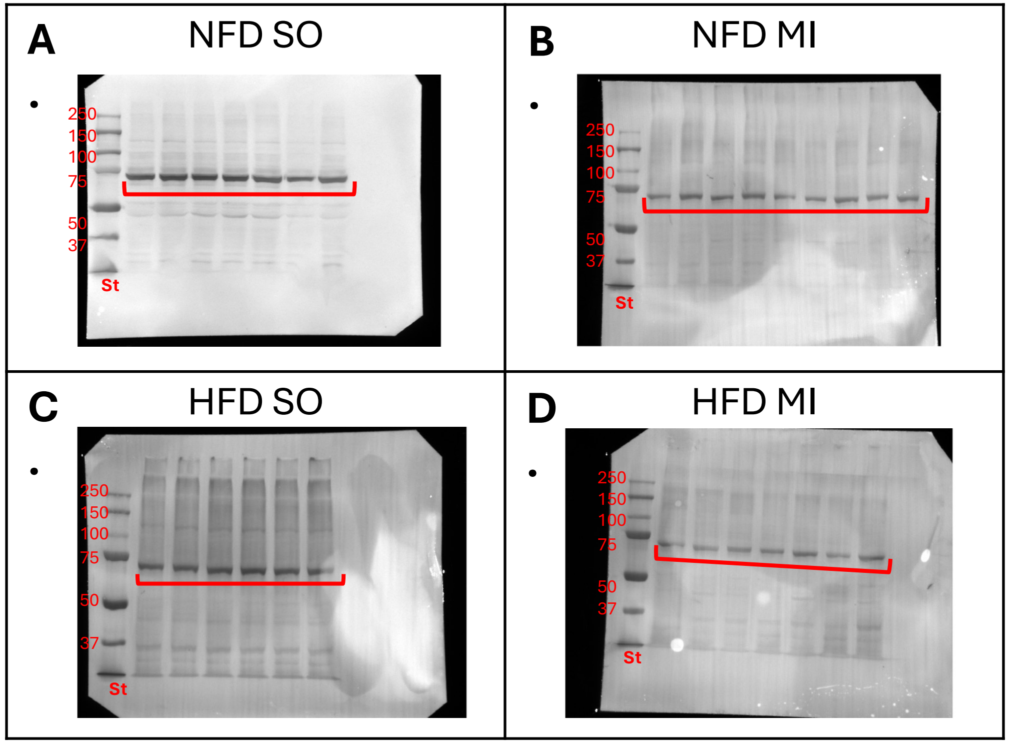

Figure 6. Western Blot results for endothelial nitric oxide syntase (eNOS). A. PVDF membrane with left ventricular samples from sham operated rats fed with the normal fat diet (NFD SO). B. PVDF membrane with left ventricular samples from rats with ligation of the coronary artery fed with the normal fat diet (NFD MI). C. PVDF membrane with left ventricular samples from sham operated rats fed with the normal fat diet (HFD SO). D. PVDF membranę with left ventricular samples from rats with ligation of the coronary artery fed with the normal fat diet. St – protein standard.

The expected size of the detected eNOS protein was around 140 kDa. However, in our results, a band was observed at approximately 75 kDa. This discrepancy might be due to differences in protein migration during electrophoresis, post-translational modifications, or protein degradation during sample preparation. We also observed additional bands at sizes other than 75 kDa, which could represent degradation products of phospho-IRE1α or non-specific antibody binding. Each sample was normalized to β-actin to minimize these differences, allowing for relative protein level comparisons between samples.

iNOS

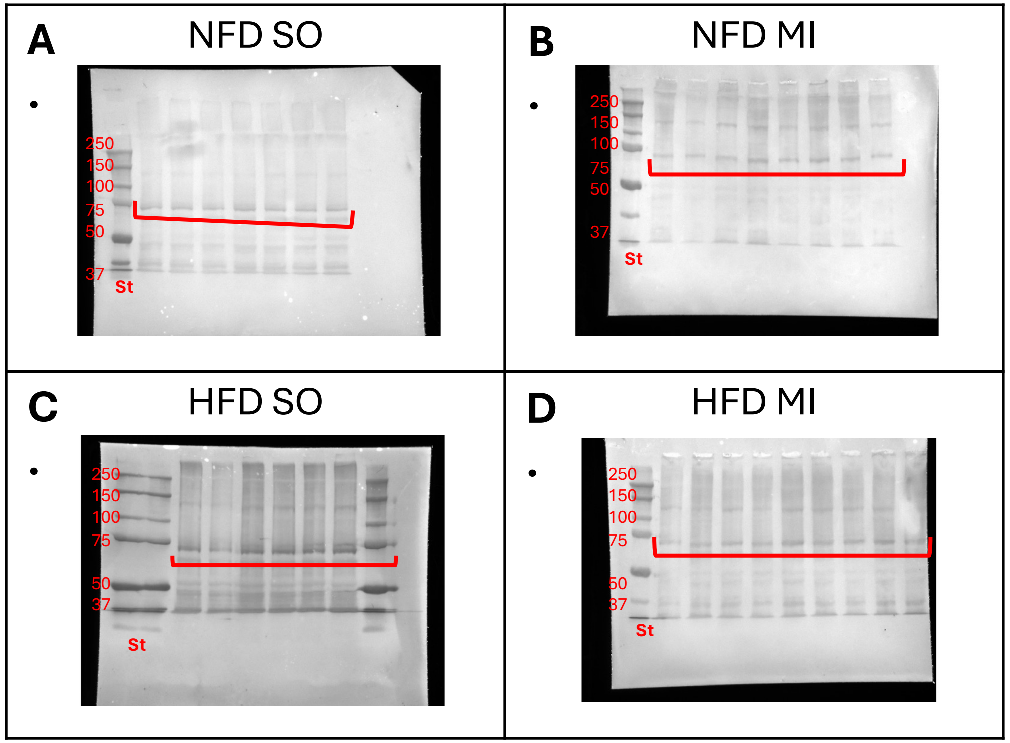

Figure 7. Western Blot results for inducible nitric oxide syntase (iNOS). A. PVDF membrane with left ventricular samples from sham operated rats fed with the normal fat diet (NFD SO). B. PVDF membrane with left ventricular samples from rats with ligation of the coronary artery fed with the normal fat diet (NFD MI). C. PVDF membrane with left ventricular samples from sham operated rats fed with the normal fat diet (HFD SO). D. PVDF membranę with left ventricular samples from rats with ligation of the coronary artery fed with the normal fat diet. St – protein standard.

The expected size of the detected iNOS protein was around 130 kDa. However, in our results, a band was observed at approximately 75 kDa. This discrepancy might be due to differences in protein migration during electrophoresis, post-translational modifications, or protein degradation during sample preparation. We also observed additional bands at sizes other than 75 kDa, which could represent degradation products of phospho-IRE1α or non-specific antibody binding. Each sample was normalized to β-actin to minimize these differences, allowing for relative protein level comparisons between samples.

nNOS

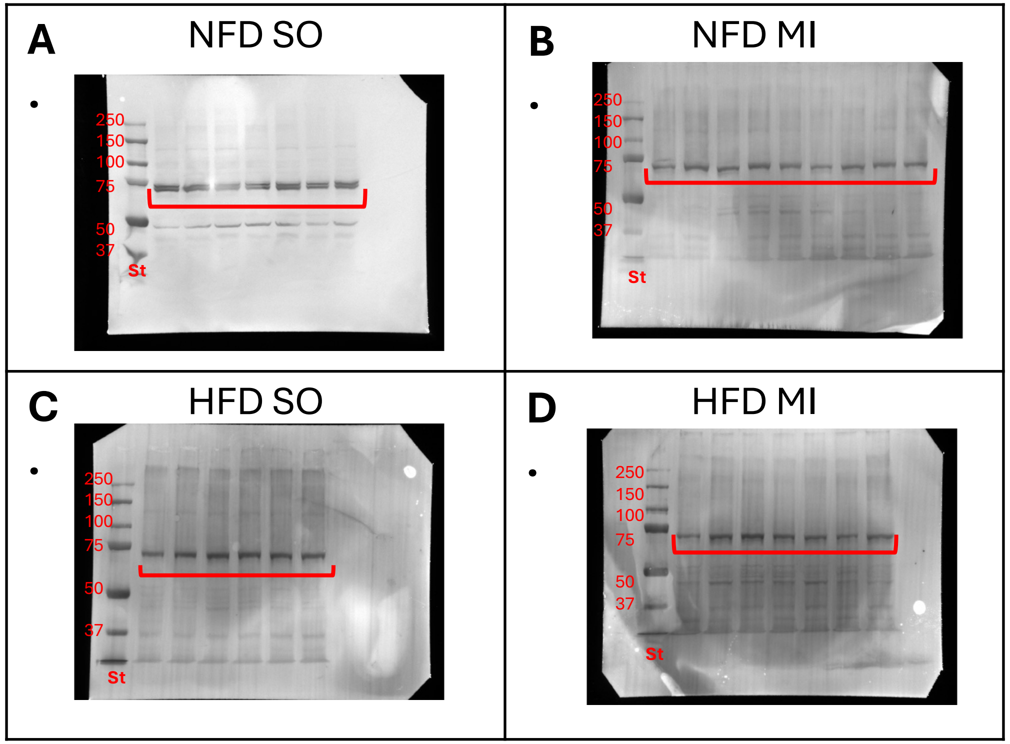

Figure 8. Western Blot results for neuronal nitric oxide syntase (nNOS). A. PVDF membrane with left ventricular samples from sham operated rats fed with the normal fat diet (NFD SO). B. PVDF membrane with left ventricular samples from rats with ligation of the coronary artery fed with the normal fat diet (NFD MI). C. PVDF membrane with left ventricular samples from sham operated rats fed with the normal fat diet (HFD SO). D. PVDF membranę with left ventricular samples from rats with ligation of the coronary artery fed with the normal fat diet. St – protein standard.

The expected size of the detected nNOS protein was around 160 kDa. However, in our results, a band was observed at approximately 75 kDa. This discrepancy might be due to differences in protein migration during electrophoresis, post-translational modifications, or protein degradation during sample preparation. We also observed additional bands at sizes other than 75 kDa, which could represent degradation products of phospho-IRE1α or non-specific antibody binding. Each sample was normalized to β-actin to minimize these differences, allowing for relative protein level comparisons between samples.

PERK

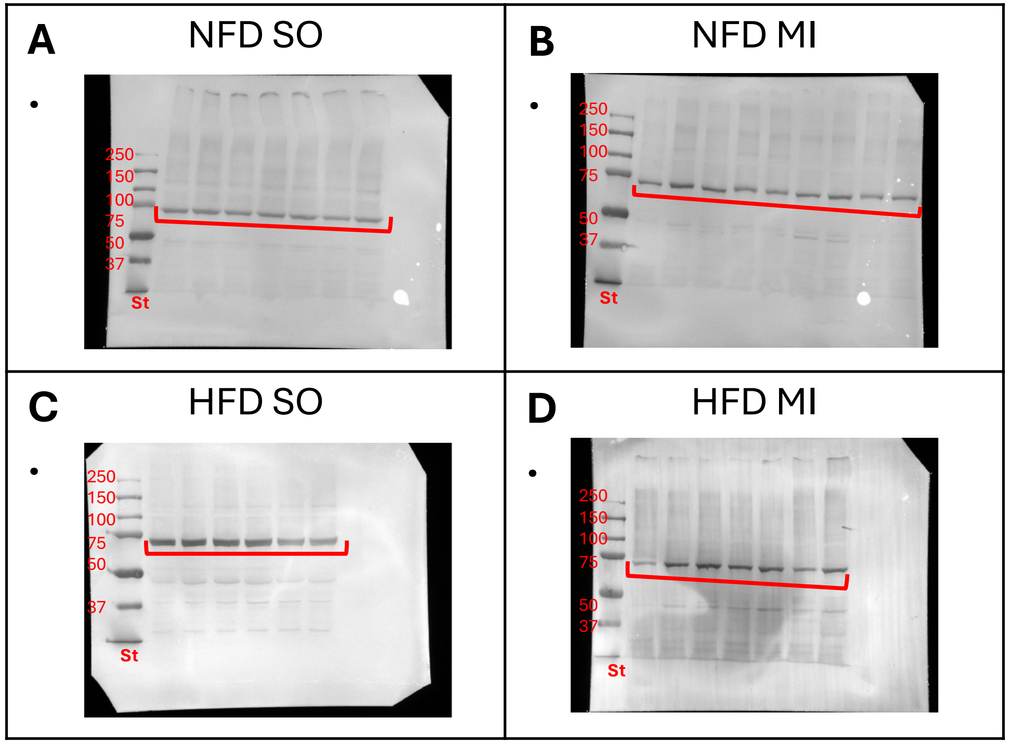

Figure 9. Western Blot results for protein kinase R-like endoplasmic reticulum kinase (PERK). A. PVDF membrane with left ventricular samples from sham operated rats fed with the normal fat diet (NFD SO). B. PVDF membrane with left ventricular samples from rats with ligation of the coronary artery fed with the normal fat diet (NFD MI). C. PVDF membrane with left ventricular samples from sham operated rats fed with the normal fat diet (HFD SO). D. PVDF membranȩ with left ventricular samples from rats with ligation of the coronary artery fed with the normal fat diet. St – protein standard.

The expected size of the detected PERK protein was around 125 kDa. However, in our results, a band was observed at approximately 70 kDa. This discrepancy might be due to differences in protein migration during electrophoresis, post-translational modifications, or protein degradation during sample preparation. We also observed additional bands at sizes other than 70 kDa, which could represent degradation products of phospho-IRE1α or non-specific antibody binding. Each sample was normalized to β-actin to minimize these differences, allowing for relative protein level comparisons between samples.

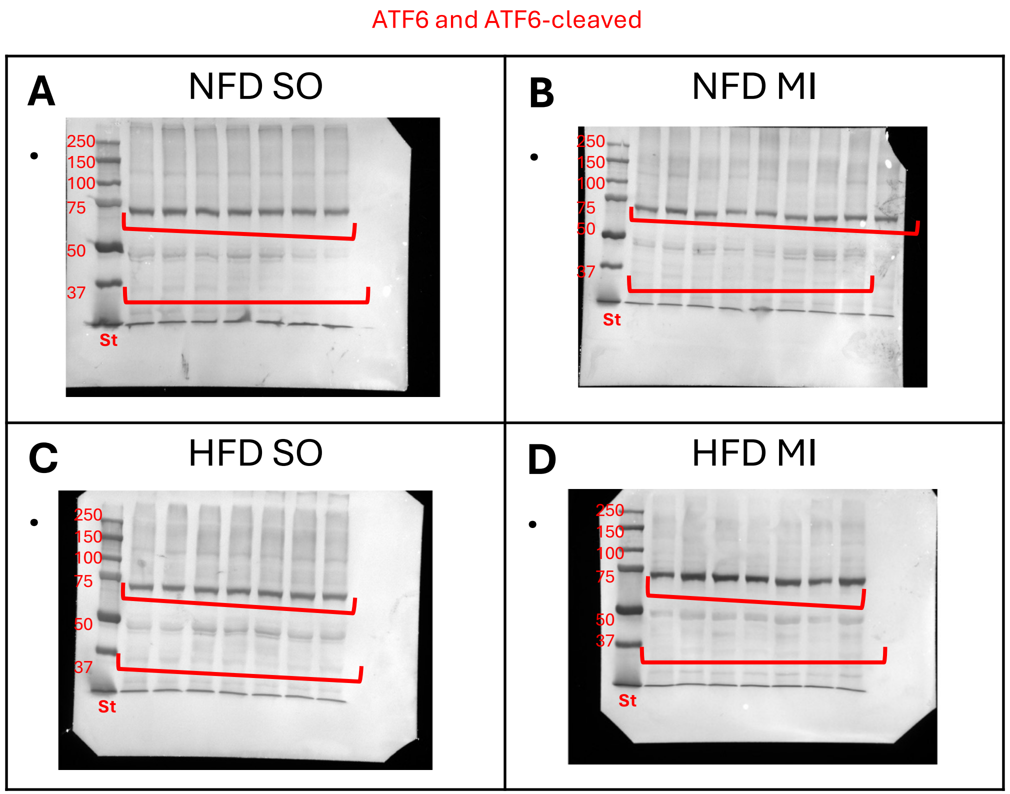

Figure 10. Western Blot results for activating transcription factor 6 (ATF6). A. PVDF membrane with left ventricular samples from sham operated rats fed with the normal fat diet (NFD SO). B. PVDF membrane with left ventricular samples from rats with ligation of the coronary artery fed with the normal fat diet (NFD MI). C. PVDF membrane with left ventricular samples from sham operated rats fed with the normal fat diet (HFD SO). D. PVDF membranę with left ventricular samples from rats with ligation of the coronary artery fed with the normal fat diet. St – protein standard.

The expected size of the detected ATF6 (non-cleaved) protein was around 90 kDa. However, in our results, a band was observed at approximately 70 kDa. This discrepancy might be due to differences in protein migration during electrophoresis, post-translational modifications, or protein degradation during sample preparation. We also observed additional bands at sizes other than 70 kDa, which could represent degradation products of phospho-IRE1α or non-specific antibody binding. Each sample was normalized to β-actin to minimize these differences, allowing for relative protein level comparisons between samples.
