# Supplementary material for: Post-myocardial infarction heart failure and long-term high-fat diet: Cardiac endoplasmic reticulum stress and unfolded protein response in Sprague Dawley rat model
Source: PLoS One. 2024 Sep 18;19(9):e0308833. doi: 10.1371/journal.pone.0308833 (PMC11410228; doi:10.1371/journal.pone.0308833)
Supplement: Attachment — Submitted filename: To the editor and reviewers.docx [file pone.0308833.s002.docx]

Dear Editor,

On behalf of all co-authors, I would like to thank the Reviewers for their valuable comments. We have addressed each comment and revised the appropriate sections accordingly. We believe these revisions have significantly improved our manuscript.

Please find our detailed responses to the specific comments below. All modifications in the text are highlighted in yellow.

Sincerely,

Karol Momot

Małgorzata Wojciechowska

**Reviewer #1:**

**The study investigated the effect of post-myocardial infarction (MI), heart failure (HF), and high-fat diet HFD on inflammation, nitro-oxidative stress, endoplasmic reticulum (ER) stress, and unfolded protein response (UPR). Twenty-eight male adult Sprague Dawley rats were divided into four groups (NFD vs. HFD) and surgical procedures (sham operation vs. coronary artery ligation to induce MI). The authors concluded that dietary fat content significantly impacts cardiac function and stress markers in a rat model of MI HF. However, several methodological weaknesses and speculative conclusions must be resolved to make the study more scientifically acceptable.**

- Thank you for your thorough review and valuable suggestions. We have addressed your comments and made the necessary revisions, as detailed below.

**• Adherence to strict dietary standards is a cornerstone of rodent research, ensuring the reproducibility and reliability of results. The dietary pattern of rodents, as outlined in AIN93 (J Nutr 1993;123:1939-51 doi:10.1093/jn/123.11.1939), is a crucial aspect that is unfortunately not detailed in the manuscript. Different types of fat can have varying effects on animal metabolism, and the protein content of the food is also vital for post-surgical recovery. Therefore, I kindly request a table providing a comprehensive breakdown of the ingredients in the control and high-fat diets used in the study.**

- We have prepared a table with detailed information about the ingredients in the normal-fat and high-fat diets used in the study, based on the manufacturer's specifications. We have also included the prepared table in the text at line 126. Thank you for highlighting this important aspect.

**• What was the difference in energy between the control and HF diets?**

- We have also included information about the caloric content of both the control and high-fat diets in the table (line 126). Thank you for your attention to this detail. This type of feed, NFD and HFD, has been used in our laboratory for many years:

1. Cudnoch-Jedrzejewska A, Gomolka R, Szczepanska-Sadowska E, Czarzasta K, Wrzesien R, Koperski L, Puchalska L, Wsol A. High-fat diet and chronic stress reduce central pressor and tachycardic effects of apelin in Sprague-Dawley rats. Clin Exp Pharmacol Physiol. 2015 Jan;42(1):52-62. doi: 10.1111/1440-1681.12324. PMID: 25311903.
2. Czarzasta K, Cudnoch-Jedrzejewska A, Szczepanska-Sadowska E, Fus L, Puchalska L, Gondek A, Dobruch J, Gomolka R, Wrzesien R, Zera T, Gornicka B, Kuch M. The role of apelin in central cardiovascular regulation in rats with post-infarct heart failure maintained on a normal fat or high fat diet. Clin Exp Pharmacol Physiol. 2016 Oct;43(10):983-94. doi: 10.1111/1440-1681.12617. PMID: 27378063.
3. Czarzasta K, Koperski L, Segiet A, Janiszewski M, Kuch M, Gornicka B, Cudnoch-Jedrzejewska A. The role of high fat diet in the regulation of MAP kinases activity in left ventricular fibrosis. Acta Histochem. 2019 Apr;121(3):303-310. doi: 10.1016/j.acthis.2019.01.010. Epub 2019 Feb 4. PMID: 30733042.
4. Czarzasta K, Wojno O, Zera T, Puchalska L, Dobruch J, Cudnoch-Jedrzejewska A. The influence of post-infarct heart failure and high fat diet on the expression of apelin APJ and vasopressin V1a and V1b receptors. Neuropeptides. 2019 Dec;78:101975. doi: 10.1016/j.npep.2019.101975. Epub 2019 Oct 15. PMID: 31645268.
5. Wojno O, Czarzasta K, Puchalska L, Kowalczyk M, Cudnoch-Jedrzejewska A. Central interaction between the apelinergic and vasopressinergic systems in the regulation of the haemodynamic parameters in rats maintained on a high-fat diet. Clin Exp Pharmacol Physiol. 2020 Dec;47(12):1902-1911. doi: 10.1111/1440-1681.13381. Epub 2020 Aug 16. PMID: 32687615.

|  | Normal-fat Diet | High-fat Diet |
| --- | --- | --- |
| Energy (kcal per 100g) | 286 | 382 |
| Carbohydrates (g per 100g) | 60.0 | 35.5 |
| Proteins (g per 100g) | 17.4 | 17.1 |
| Fats (g per 100g) | 3.2 | 28.0 |
| Saturated (%) | 13 | 49 |
| Unsaturated (%) | 37 | 44 |
| Polyunsaturated (%) | 50 | 7 |
| Raw ash (g per 100g) | 12 | 12 |
| Water (g per 100g) | 12 | 12 |

**• The names of groups should be more explicit and not confusing.**

- We have changed the group names from HFD HF to HFD-MI and NFD HF to NFD-MI to make them more explicit and less confusing. All the changed names have been highlighted in yellow. Thank you for your suggestion.

**• Page 5 - Fig 1 is mentioned, but we see a table.**

- At this point in the text, both Figure 1 and Table 1 should be included. Table 1 is included in the text, and following the author guidelines, figures should not be placed within the main text. Figure 1 is included in the attachments.

**• In line 103, three references are cited to explain the experimental procedures (refs. #24-26). However, these references are a meta-analysis and two reviews that do not provide experimental details.**

- The appropriate references are now #29-31, which were initially listed at the bottom of the reference list (Line 104). Apologies for the error.

**• Coronary artery ligation in rodents is not always lethal, but many animals die. The manuscript does not inform how many animals were operated on or the survival rate.**

- We have added information about the number of animals operated on and the survival rates in the main text:
  - Sham-operated and High-fat Diet: 10 rats operated, 6 survived
  - Coronary artery ligation and High-fat Diet: 20 rats operated, 10 survived
  - Sham-operated and Normal-fat diet: 10 rats operated, 7 survived
  - Coronary artery ligation and Normal-fat diet: 15 rats operated, 9 survived

Please see Table 1, line 110.

**• When ligating the coronary artery to cause a myocardial infarction in rodents, the method produces individual lesions; some animals may have an extensive infarction area, but others may not. Evans blue and triphenyl tetrazolium chloride staining can determine infarct areas at sacrifice (a missing item that compromises the study).**

- We measured the infarct area using the planimetric method: the HFD-MI group had a significantly larger infarction area than the NFD-MI group (44.57 ± 3.44% vs. 25.29 ± 0.75%, P< 0.001). We did not use Evans blue and triphenyl tetrazolium chloride staining because post-euthanasia, we collected the heart (both right and left ventricles) for gene expression analysis using RT-PCR or to estimate protein levels using Western Blot or ELISA. Staining the hearts could have affected the results of these biochemical analyses or rendered them impossible. Therefore, we opted for the planimetric method to assess the infarct scar.

**• Did the operated animals eat normally? How was the animals' food intake? The information is relevant to understanding the result of body mass loss reported in the article.**

- We did not measure the food intake of the operated animals. However, we did assess fat tissue masses, which can be found in the articles cited in our main text:

1. Czarzasta K, Cudnoch-Jedrzejewska A, Szczepanska-Sadowska E, et al. The role of apelin in central cardiovascular regulation in rats with post-infarct heart failure maintained on a normal fat or high fat diet. Clin Exp Pharmacol Physiol. 2016;43(10):983-94. <https://doi.org/10.1111/1440-1681.12617>
2. Czarzasta K, Wojno O, Zera T, et al. The influence of post-infarct heart failure and high fat diet on the expression of apelin APJ and vasopressin V1a and V1b receptors. Neuropeptides. 2019;78:101975. <https://doi.org/10.1016/j.npep.2019.101975>

**• Line 139 –reference #24 used to describe the protein analysis method is a meta-analysis, so…**

- The situation is the same as in line 103. We apologize for the mistake. The correct citation should be #29. Thank you for your attention to detail. Please see line 143.

**• I did not find an explanation for what proBNP means (which appears a few times in the text).**

- We have added an explanation for the abbreviation proBNP in the text. Please see line 132-133. Thank you for pointing this out.

**• Lines 206-2010 - Nitric oxide (NO) is a gas that degrades quickly. The study did not evaluate NO production and cannot discuss NO levels.**

- We agree with the Reviewer’s opinion. However, in our study, we analyzed the levels of synthases, which are relatively stable proteins and do not degrade as quickly as NO. These can be analyzed using Western blot.

**• Comparisons between groups (and markings on graphs) - there are comparisons between groups that are not comparable (e.g., unoperated ND vs. operated HFD) and should not be stated or commented on.**

- Thank you for your observation. We have corrected the figures and the text to ensure only comparable groups, which differ by a single variable (either the type of procedure or the diet, but not both simultaneously), are stated and commented on..

**• The statistical analysis should be more explicit and inform where the parametric or non-parametric analysis was performed. Why was the Spearman coefficient not determined for non-parametric correlations?**

- We did not use the Spearman coefficient because each analyzed data point followed a normal distribution and exhibited a linear relationship. Therefore, we used only the Pearson method. Additionally, upon closer analysis, we confirmed that all quantitative data were analyzed using one-way ANOVA, and none with the Kruskal-Wallis test. We have added a note about this in the methodology section. See lines 158-164. Thank you for your suggestion and apologies for the confusion.

**• I do not understand how the authors talk about 'heart failure' (HF) if the study does not include measuring the animals' blood pressure or a cardiac functional test (ultrasound?). Analysis of left ventricular ejection fraction is essential to understand the effect of post-infarction HFD.**

- We assessed the infarct area using the planimetric method and measured NT-proBNP levels in plasma to determine if the rats had developed post-infarction heart failure. Thank you for highlighting the importance of these measurements.
- In a separate study, we evaluated hemodynamic parameters, including left ventricular end-diastolic pressure (LVEDP), using invasive catheterization in both infarcted and non-infarcted rats on high-fat or standard diets:
  - Czarzasta K, Cudnoch-Jedrzejewska A, Szczepanska-Sadowska E, et al. The role of apelin in central cardiovascular regulation in rats with post-infarct heart failure maintained on a normal fat or high fat diet. Clin Exp Pharmacol Physiol. 2016;43(10):983-94. <https://doi.org/10.1111/1440-1681.12617>).

**• There are numerous grammatical and spelling errors in the text.**

- Thank you for pointing this out. We have thoroughly reviewed the text again and corrected all grammatical and spelling errors.

**Reviewer #2:**

**This manuscript describes an exploratory study examining the influence of high fat diet on cardiac nitrosative stress and endoplasmic reticulum stress in rat model with post-myocardial infarction heart failure. Overall, the manuscript is well written.**

- Thank you very much for taking the time to review our manuscript. We have addressed your comments below.

**Although the novelty of this experiment is limited by the great depth of literature available using other rodent models, this manuscript reports a technically-sound experiment describing the nitrosative and endoplasmic reticulum stress markers in rats.**

- Thank you for your feedback. As this was a preliminary study and the results were very interesting, we decided to submit this article to the journal. To date, no one has analyzed nitrosative stress in the context of myocardial infarction and a high-fat diet. It is known that this stress is exacerbated in heart failure with preserved ejection fraction. Publishing these results will lay the foundation for further research and inspire more researchers to explore this topic.

**Nonetheless, the manuscript lacks any functional metrics that could support and verify the development of heart failure in this model.**

- We assessed the infarct area using planimetric methods and measured NT-proBNP levels in plasma to determine if the rats had developed post-infarction heart failure. In a separate study, we also evaluated hemodynamic parameters, including LVEDP (left ventricular end-diastolic pressure) using invasive catheterization in both infarcted and non-infarcted rats on high-fat or standard diets (refer to Table in Experiment 1 from DOI: 10.1111/1440-1681.12617).

**The choice of single time-point also limits the information provided by this characterization study whereby it is unclear when does nitrosative stress and endoplasmic reticulum stress occurs with diet and MI-induced heart failure.**

- Thank you for your insightful feedback. We would like to emphasize that these are preliminary studies, and extending the study to include additional time points is challenging due to the high mortality rate of rats following the ligation of the left coronary artery. Specifically, we observed the following survival rates in our study (See Table 1, line 110):
  - Sham-operated and High-fat Diet: 10 rats operated, 6 survived
  - Coronary artery ligation and High-fat Diet: 20 rats operated, 10 survived
  - Sham-operated and Normal-fat diet: 10 rats operated, 7 survived
  - Coronary artery ligation and Normal-fat diet: 15 rats operated, 9 survived

We chose to include one control group and three experimental groups in our study. This design allowed us to use two-way ANOVA analysis to evaluate the impact of different factors on various parameters of stress and inflammation
