# Supplementary material for: Post-myocardial infarction heart failure and long-term high-fat diet: Cardiac endoplasmic reticulum stress and unfolded protein response in Sprague Dawley rat model
Source: PLoS One. 2024 Sep 18;19(9):e0308833. doi: 10.1371/journal.pone.0308833 (PMC11410228; doi:10.1371/journal.pone.0308833)
Supplement: Attachment — Submitted filename: To the reviewer 2.docx [file pone.0308833.s003.docx]

Dear Reviewer,

Thank you for your feedback and suggestions. We have made the necessary revisions to the text in response to reviews and have emphasized that this study is preliminary research in both the discussion and conclusion sections.

For your review, the changes have been made using the track changes mode.

We sincerely thank both reviewers for the valuable and scientific discussion. Your input has been greatly appreciated and has significantly contributed to the development of our study. We hope this clarification addresses your concerns.

Sincerely,

Karol Momot and colleagues
